# Cognitive Performance and Diabetic Retinopathy: What Your Eyes Can Reveal About Your Brain

**DOI:** 10.2174/1573399819666220805154638

**Published:** 2023-08-02

**Authors:** Ana Cristina Ravazzani de Almeida Faria, Joceline Franco Dall'Agnol, Aline Maciel Gouveia, Clara Inácio De Paiva, Victoria Chechetto Segalla, Fernando Eiji Ogata, Cristina Pellegrino Baena

**Affiliations:** 1 Postgraduate Program in Health Sciences, Pontifical Catholic University of Paraná, (PUCPR), Curitiba, Paraná, Brazil;; 2 Department of Medicine, Pontifical Catholic University of Paraná (PUCPR), Curitiba, Paraná, Brazil;; 3 Hospital of Eyes of Paraná, Curitiba, Paraná, Brazil

**Keywords:** Diabetic retinopathy, cognitive dysfunction, type 2 diabetes mellitus, risk factors, dementia, cognitive decline

## Abstract

**Background:**

Diabetic retinopathy (DR) is a chronic diabetes complication. People with Type 2 Diabetes Mellitus (T2DM) have two times the risk for dementia, suggesting it is a new chronic diabetes complication.

**Objective:**

Evaluate the association of DR with cognitive performance in a T2DM population.

**Methods:**

Cross-sectional study with 400 T2DM adults from whom socio-demographic, clinical, laboratory data were collected, and screening test for depression symptoms (Patient Health Questionaire-9 (PHQ-9)), Mini-Mental State Examination (MMSE), Semantic Verbal Fluency Test, Trail Making Test A and B, Word Memory test were performed. All cognitive test scores were converted into Global Cognition z-Score (GCS(z)). The association between GCS(z) < 0 with DR was performed using a multivariate binary logistic regression model adjusted for age ≥ 65 years, school years ≤ 6 years, DM duration ≥ 10 years, depression symptoms score > 9 at PHQ-9, arterial hypertension, physical activity, diabetic retinopathy, macular edema, and cardiovascular disease.

**Results:**

After exclusions, the 251 eligible patients were 56.6% female, with a mean age of 61.1 (±9.8) years, DM duration of 12.6 (±8.9) years, and 7.6 (±4.2) years of school education. DR prevalence was 46.5%. Multivariate Logistic Regression Model showed an association between DR and GCS(z) < 0, with odds ratio (CI95%) of 2.50 (1.18-5.34), adjusted for age, low education level, arterial hypertension and depression symptoms (OD and CI95% respectively: 5.46(2.42-12.34); 12.19 (5.62-26.46); 2.55 (0.88-7.39); 3.53 (1.55-8.07)).

**Conclusion:**

In this T2DM population, having DR increased the chance for worse cognitive performance even when adjusted for age, low education level, presence of arterial hypertension, and depression symptoms.

## 1 INTRODUCTION

Type 2 Diabetes Mellitus has long-term micro and macrovascular complications related to DM duration, glycemic control, and comorbidities. Among these complications, diabetic retinopathy (DR) is, according to the World Health Organization, responsible for 4.8% of blindness cases in the world. A meta-analysis of 35 studies with 22,896 people with Type 1 and 2 DM, evaluated between 1980 and 2008, in the United States of America, Australia, Europe,and Asia, found a global prevalence of DR of 34.6% and critical loss of visual acuity of 10.2%. For type 2 DM alone, DR prevalence was 25.2%, and critical loss of visual acuity was 6.9% [1-4]. Longitudinal studies identified that the risk for dementia is greater in the population with diabetes [5-12]. This risk is 1.73 for all types of dementia when compared to people without diabetes [12, 13]. The mechanisms by which DM acts as a casual or accelerating factor in the dementia process, whether vascular dementia or Alzheimer’s dementia, are not yet fully known. Several pathogenic mechanisms have been postulated, among them: chronic hyperglycemia and its enzymatic glycation end products, insulin resistance, glycemic variability, hypoglycemia, DM microvascular disease, cardiovascular disease and its risk factors, in addition to inflammation, oxidative stress, and metabolism of amyloid peptides, amylin and tau protein [14-16]. Microvascular dysfunction is well recognized as part of DM complications, including cerebral microvascular dysfunction. The genesis of microvascular dysfunctions is associated with arterial hypertension, hyperglycemia, obesity, and insulin resistance [17]. Among the classic DM microvascular complications, DR has been identified in cross-sectional and longitudinal studies as a risk factor for the presence of dementia and cognitive dysfunction in patients with Type 1 and 2 DM [18-21]. It seems logical to think that micro and macrovascular alterations that occur in other parts of the body happen similarly in the brain. According to this logic, the retinal microvascular evaluation would mirror the brain microvasculature since the retina and the brain have very similar anatomy, embryology, and physiology. A review of studies that evaluated the association between retinal vascular alterations and dementia or cognitive deterioration found an association between retinal alterations and these outcomes, as well as with the alterations in brain imaging. This association was greater as the retinal alterations were more severe and suggested vascular pathophysiology, but the effect size was modest, possibly due to the concurrence of other associated risk factors [22]. On the other hand, another populational study showed an association between retinal neurodegeneration and the presence of cerebral atrophy and not with cerebral vascular alterations, suggesting that this association may be due to neuronal degeneration and not to vascular dysfunction [23]. Therefore, the mechanisms of these associations are not yet fully clear, and further studies are necessary to better understand the pathophysiology of this association and identify whether vascular or retinal neurodegeneration markers can be used as predictors of cognitive dysfunction in this population, considering that until now, there is still no specific biomarker or set of biomarkers, as well as no imaging exam to determine future risk of minimal cognitive dysfunction (MCD) and dementia or to determine the ones who will have a worse prognosis [24]. This study aimed to assess the association of DR and macular edema with cognitive performance in a population of T2DM patients in an upper-middle-income country.

## 2 MATERIALS AND METHODS

### 2.1 Study Design and Sample

A cross-sectional study was conducted in a tertiary hospital in Southern Brazil, from September 2017 to December 2020, with patients over 18 years old with T2DM of both genders, randomly recruited according to their attendance at their routine appointments. Patients with T2DM were considered those who did not need insulin in the first 3 years of the disease and had no history of ketonuria or ketonemia at diagnosis [25]. Patients who were using medicines that alter cognition (benzodiazepines, hypnotics, antipsychotics, tricyclic antidepressants, anticonvulsants, anticholinergics, and antihistamines) were excluded, as well as those who were unable to perform the cognitive tests due to illiteracy, vision or hearing impairment. Those with a previous diagnosis of dementia of any etiology, stroke, traumatic brain injury, Parkinson's disease, schizophrenia, or any other situation that affects cognition were also excluded, as well as the ones that met dementia criteria at the MMSE test. The study was approved by the Research Ethics Committee and conducted following the principles of the Declaration of Helsinki [26].

### 2.2 Data Collection

Participants answered a questionnaire containing demographic data (age, gender, race, marital status, and school education), lifestyle data (physical activity, alcohol consumption, smoking), and medical history [DM onset age, acute complications (severe hypoglycemia) and diabetes chronic (retinopathy, neuropathy, diabetes kidney disease, cardiovascular disease)], comorbidities and use of medication. Additional information was captured from medical records. It was considered physically active participants who met the criteria of at least 150 minutes of moderate or 75 minutes of intensive aerobic exercise per week. Severe hypoglycemia was defined as that in which the patient needed help from others for treatment and/or had a decrease in consciousness level, with improvement in symptoms after treatment [27]. The following data was collected from the physical examination: Body Mass Index (BMI), Abdominal and Neck Circumference, Systolic Blood Pressure (SBP), and Diastolic Blood Pressure (DBP). Arterial hypertension diagnosis was defined as SAP ≥ 140 mmHg and DBP ≥ 90 mmHg or also if using antihypertensive medication [28]. Retina clinical examination was performed at the ophthalmologic clinic by retinal mapping under drug-induced mydriasis by indirect binocular ophthalmoscopy and slit-lamp biomicroscopy and, when indicated, by fluorescein angiography and optical coherence tomography classified as no diabetic retinopathy (DR), non-proliferative DR, proliferative DR, and macular edema [25, 29, 30]. Diabetic neuropathy was considered in the presence of clinical symptoms and signs compatible with peripheral sensory-motor neuropathy according to the guidelines of the Brazilian Diabetes Society, based on peripheral neurological clinical examination [25]. Laboratory tests and complementary tests data were retrieved from medical records: glycated hemoglobin a1c (HBA1c), fasting glucose, total cholesterol, low-density lipoprotein (LDL), high-density lipoprotein (HDL) cholesterol, triglycerides, thyroid-stimulating hormone, free thyroxine, B12 vitamin, creatinine, and albumin-to-creatinine ratio (ACR). Creatinine value was used to calculate the estimated glomerular filtration rate (eGFR) adjusted for age and gender using the CKD-EPI (Chronic Kidney Disease Epidemiology Collaboration) formula [25, 27, 31, 32]. DM kidney disease was considered in the presence of eGFR < 60ml/min/1.73m^2^ or ACR > 30 mg/g persistently elevated for more than 3 months, following the recommendations of KDIGO (Kidney Disease: Improving Global Outcomes) and the guidelines of the Brazilian Diabetes Society [25, 33]. Diagnosis of dyslipidemia was based on the Guidelines of the Brazilian Cardiology Society (LDL ≥100 mg/dl if intermediate cardiovascular risk, ≥ 70 mg/dl if high cardiovascular risk; HDL ≤ 45 mg/dl; Triglycerides ≥ 150 mg/dl or using lipid lowering medication) [34].

### 2.3 Cognitive and Depression Symptoms Assessment

The cognitive tests performed, validated in Portuguese, were: Trail Making Test A and B to assess sustained attention, mental flexibility, executive function, spatial/visual organization, and processing speed [35-38]; Semantic Verbal Fluency test to assess semantic memory storage capacity, ability to retrieve information from memory, and processing of executive functions [37-40]; CERAD (The Consortium to Establish a Registry for Alzheimer’s Disease) Word Memory Test to assess memory [38-40] and Mini-Mental State Examination, to screen patients with dementia and make a global assessment of cognition, covering aspects of orientation, memory, attention, calculation, language, and comprehension [32, 35-41]. The cutoff value of this test varies according to education level, and scores with values below the cutoff corrected by the education level in the Brazilian population were used to indicate the risk of dementia [42-47] (Supplementary file **1**). The PHQ-9 (Patient Health Questionaire-9), validated in Portuguese, was carried out and values above 9 were considered as a risk of a diagnosis of major depression (Supplementary file **2**) [48, 49].

### 2.4 Statistical Analysis

To uniformly analyze the set of different cognitive tests, the results of all tests were transformed into Z scores, added, and divided by the total number of tests. This result created a continuous variable called Global Cognitive Score [GCS(z)]. A GCS(z) < 0 at baseline means a worse cognitive performance compared to the group. The association estimation of GCS(z) < 0 with DR adjusted for risk factors for cognitive dysfunction was performed using backward conditional multivariate binary logistic regression. The categorized variables chosen to be included in the model were: age 65 years (yes/no), school education 6 years (yes/no), gender (female/male), physically activity (yes/no), smoking (current or previous/never) alcoholism (current or previous/never), severe hypoglycemia (yes/no), DM 10 years (yes/no), PHQ-9 > 9 (yes/no), arterial hypertension (yes/no), depression/anxiety diagnosis (yes/no), cardiovascular disease (yes/no), use of insulins (yes/no), use of statins (yes/no), hypothyroidism (yes/no), any DR (yes/no), macular edema (yes/no), diabetic neuropathy (yes/no), DM kidney disease(yes/no), BMI ≥ 30 kg/m^2^ (yes/no), eGFR < 60 ml/min/1.73 m^2^ (yes/no) and HBA1c ≥ 7%(yes/no). Variables with p < 0.25 in the univariate binary regression analysis were used in the final model [44]. For all other tests, the significance level used was 5% (SPSS version 22. IBM Corporation, Armonk, NY^®^) [50].

## 3 RESULTS

**Fig. (1):**
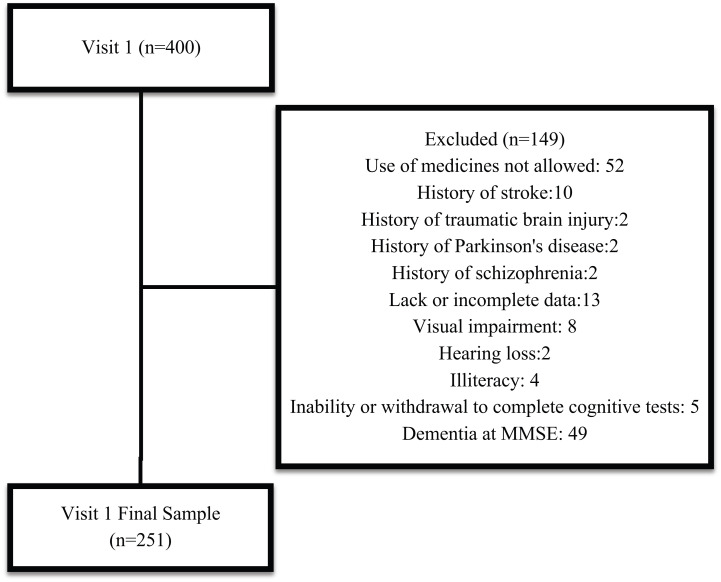
Sample flux diagram.

Among the 400 patients evaluated, 149 were excluded (Fig. **1**).

The final sample consisted of 251 patients, 56,6% female, with mean age of 61,1 (±9,8) years, 12,6 (±8,9) years of DM duration, and 7,6 (±4,2) years of school education. Two hundred and six patients (82,4%) were hypertensive and 89% had dyslipidemia. The prevalence of at least one microvascular complication was 54%, with DR being prevalent in 46,5%. Cardiovascular disease was present in 35,2%. In the screening for depression symptoms by the PHQ-9, 37,1% had a score compatible with the risk of major depression and 21,3% had presented at least one episode of severe hypoglycemia in the previous year. DBP value was controlled in approximately 36% of the patients and SBP in 65% (Table **1**). Data from laboratory tests were within normal ranges, except for lipid profile, fasting glucose, and HBA1c, with only 30% having HBA1c < 7%. LDL cholesterol levels were within expectations in about 21% of patients and HDL cholesterol in 40%, according to individual stratification of individual risk (Table **1**). The prevalence of GCS(z) < 0 was 46,6% and the summary of cognitive tests and GCS(z) results are described in Table **1** (Supplementary Files **3** and **4**).

**Fig. (2):**
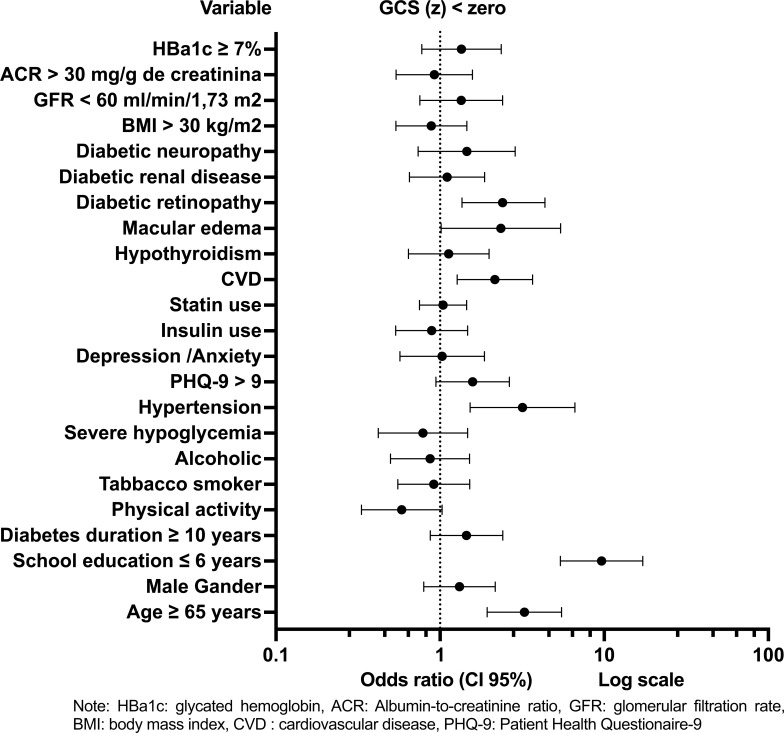
Predictive factors for Global Cognitive Score < 0 Univariate Logistic Regression.

In the univariate binary logistic regression model, variables identified as significantly associated with GCS(z) < 0 and included in the multiple regression model were: age ≥ 65 years, school education 6 years, DM duration ≥ 10 years, physical activity, score on the PHQ-9 questionnaire > 9, arterial hypertension, cardiovascular disease, DR, and macular edema (Fig. **2**) (Supplementary Files **5**).

**Fig. (3):**
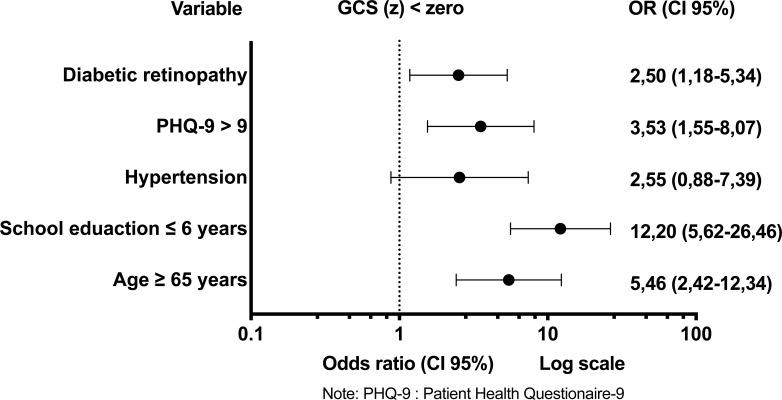
Predictive factors for global cognitive score < 0. Multivariate logistic regression.

The multivariate logistic regression model showed an association between DR and GCS(z) < 0, with odds ratio (OD) and 95% confidence interval (CI95%) of 2,50 (1,18-5,34); even after adjustment for age ≥ 65 years, school education years ≤ 6, arterial hypertension and depression symptoms [OD and CI95% respectively: 5,46(2,42-12,34); 12,19(5,62-26,46); 2,55(0,88-7,39); 3,53(1,55-8,07)] (Fig. **3**) (Supplementary File **6**).

## 4 DISCUSSION

In this sample, DR was associated with worse cognitive performance, regardless of advanced age, low education level, arterial hypertension, and depression symptoms.

Age and schooling are well-known and expected MCD and dementia risk factors [51-54]. In this sample, schooling was the risk factor that most contributed to a chance of scoring below average on the GCS(z), followed by age. The Brazilian Longitudinal Study of Adult Health (ELSA-Brazil) identified that education, more than age, interferes with the results of the cognitive tests in one Brazilian population. Another regional study that assessed cognitive performance in a population of DM patients found the same association between the MMSE results, education, and age [47]. In the last Dementia Prevention, Intervention and Care Commission report, published in the Lancet magazine in 2020, school education lower or equal to 4 years was identified as a relative risk of 1.6 for the development of dementia and predicted that if only this risk factor could be eliminated, the prevalence of dementia would be reduced by 7% [55]. These findings reinforce the need to assess the results of cognitive tests according to education and age, or at least adjust them for these variables.

The retina and the brain have the same embryological origin and similar pathophysiological and aging mechanisms. Therefore, it can be an easily accessible source of information for cerebral neurodegenerative processes. Communication between the brain and the retina occurs through retinal ganglion cells, connecting with the cortex through the optic nerve. The blood-brain and blood-retinal barriers regulate the supply of oxygen and glucose to the neurons and prevent the exposure of the central nervous system to pathogens and toxic substances, thus protecting the central nervous system and retina microenvironment, respectively. Among other functions, the barrier tries to protect neurons from inflammatory cytokines commonly circulating in patients with diabetes and its comorbidities [56, 57]. The production or activation of inflammatory cytokines at the brain level can lead to insulin action resistance in the brain, resulting in deterioration of brain processes such as neuron survival, dendritic plasticity, synaptic function, learning, and memory [58-60].

Optical coherence tomography (OCT) is a non-invasive exam that allows the measurement of retinal thickness and its components. The retinal nerve fiber layer is the innermost part of the retina and is formed by retinal ganglion cells and alterations in this region are associated with neurodegeneration [20, 61, 62]. The Rotterdam cohort study found a correlation between neurodegenerative retinal alterations detected in OCT with brain atrophy, but not with micro-hemorrhages or lacunar infarcts in the nuclear magnetic resonance [23]. Retinal glial, neural, and microvascular dysfunction are interdependent for the development of diabetic retinopathy. A thinner retinal nerve fiber layer thickness is present even with minimal DR in T1DM, possibly anteceding the retina's vascular deterioration [15, 63]. Other studies have observed an association between corneal neurodegeneration findings with cognitive alterations and imaging findings related to MCD and dementia [64, 65].

Retinal microcirculation can also be non-invasively visualized using retinal arteriography and its alterations have also been associated with cognitive decline in Type 1 and 2 DM populations with long-term duration [18, 20, 21, 66-68]. Therefore, both, microcirculation and neurodegenerative retinal and corneal alterations are associated with cognitive changes and dementia, but the mechanisms supporting this association are not fully elucidated.

Visual loss on its own may also be a risk factor or be associated with MCD and dementia. A meta-analysis of prospective studies in the general population showed that moderate and severe loss of visual acuity is associated with cognitive dysfunction. However, evidence quality is low and other studies disagree on this topic. Therefore, it is still necessary to confirm this possible association [69-74].

Recently a systematic review and meta-analysis of twenty-two studies, including cross-sectional and cohort studies showed a similar association between DR and cognitive impairment. In this study, the presence of DR reflected a higher cognitive dysfunction with OR=2,45(95%CI:1,76-3,41) and HR=1,34(95%CI:1,10-1,62). The pooled OR was 2,38 and 3,11 for Asia and Oceania respectively, and there was no association in North America and with T1DM. There was no study from South America. They also found that DR severity showed a positive correlation with cognitive impairment [75]. One other review and meta-analysis evaluated the association between DR and cerebral small vessel disease with any type of cognitive dysfunction and found an association between DR and structural abnormalities in the brain and impaired cognitive function [76].

Ophthalmologic evaluation is already part of routine screening for complications related to DM and is recommended at diagnosis and once a year thereafter. Further exploring the association of retinal alterations in the DM population and the ability to potentially predict present and future cognitive performance may be a useful tool for selecting higher-risk patients for evaluation and follow-up.

One limitation of this study is the absence of assessment of biomarkers, retinal neurodegeneration images, and brain images to correlate with clinical data. Most patients only underwent retinal angiography, and none had brain images. However, we reproduced data from a real-life scenario in public health setting that could be generalized for similar health services in developing countries. In addition, these findings should be monitored to confirm their long-term role.

## CONCLUSION

There are few studies in Brazil evaluating risk factors related to cognitive performance and dementia in the population with DM, and none of them has explored the presence of retinopathy as a risk factor or associated factor. This study showed this association even after adjustment for other important risk factors. The association between DR and MCD in this population is an interesting field to be explored, as it has the potential to select patients at risk through simple and non-invasive exams during the evaluation of a complication that is already part of the medical routine of people with DM, avoiding more costs and more procedures. The evolution of science towards finding biomarkers and/or image markers to help predict cognitive evolution in middle-aged patients with T2DM should be valuable for assessing cognition in this population. Neurodegeneration and retina vascular markers may be promising for this function. Meanwhile, seeking clinical markers that can help in the early identification of higher-risk patients for cognitive alterations may be useful to try to delay cognitive decline in this population.

## Figures and Tables

**Table 1:** Sample characteristics.

**Characteristics**	**Mean** ± SD / Median (IQR)*/%
**Age (years)**	61.1 ± 9.8
**School education (years)**	7.6 ± 4.2
**DM duration (years)**	12.6 ± 8.9
**Female (%)**	56.6
**Physically Active (%)**	27.9
**Smoker/Former Smoker (%)**	47.1
**Alcoholic/Former alcoholic (%)**	29.5
**Diastolic Blood Pressure (mmHg)**	80.4 ±10.8
**Systolic Blood Pressure (mmHg)**	131.2 ± 17.8
**BMI (Kg/m^2^)**	30.8 ± 5.3
**Arterial hypertension (%)**	82.4
**Dyslipidemia (%)**	89.0
**Hypothyroidism (%)**	26.1
**Cardiovascular disease (%)**	35.2
**Diabetic Retinopathy (%)**	46.5
**Macular edema (%)**	13.8
**Diabetic Neuropathy (%)**	16.1
**Diabetic kidney disease (%)**	54.0
**Severe hypoglycemia (%)**	21.3
**Depression/Anxiety (%)**	22.8
**PHQ-9 score > 9 (%)**	37.1
**Insulin Use (%)**	58.8
**Statins Use (%)**	76.2
**eGFR (ml/min/1.73m^2^)**	84.8(36.3)
**HbA1c (%)**	8.0(2.4)
ACR (mg/g creatinine)*	21.4(80.6)
MMSE (score)*	27.2 ± 2.0
**Verbal fluency (score)**	16.5 ± 4.9
**TMT A (seconds)**	56.3 ± 27.9
**TMT B (seconds)**	162.8 ± 107.7
**Immediate Memory (score)**	16.3 ± 4.5
**Recall Memory (score)**	5.1 ±1.9
**Recognition Memory (score)**	8.1 ± 2.0
**GCS(z) (score)**	-0.015 ± 0.671
**GCS(z) < 0 (%)**	46.6

**Note:** SD: Standard deviation: * IQR: Interquartile range

DM: *Diabetes Mellitus:* BMI: Body Mass Index: PHQ-9: Patient Health Questionnaire-9, eGFR: estimated Glomerular Filtration Rate, ACR: Albumin-to-creatinine ratio, TSH: thyroid-stimulating hormone, MMSE: Mini-Mental State Exam, TMT A and B: Trail making test A and B: GCS(z), Global Cognitive score (z).

## Data Availability

Most data generated or analyzed during this study are included within the article and its supplementary information file. Any additional data is available from the corresponding author [CPB] on reasonable request.

## LIST OF ABBREVIATIONS

HBA1c: Glycated hemoglobin
DM: Diabetes mellitus
T2DM: Type 2 Diabetes mellitus
MCD: Minimal cognitive decline or dysfunction
CVD: Cardiovascular disease
GCS(z): Global cognitive score
HDL: High-density lipoprotein
BMI: Body mass index
LDL: Low-density lipoprotein
MMSE: Mini-Mental State Examination
DBP: Diastolic blood pressure
SBP: Systolic blood pressure
PHQ-9: Patient Health Questionaire-9
ACR: Albumin-to-creatinine ratio
DR: Diabetic Retinopathy
eGFR: estimated Glomerular filtration rate
OCT: Optical coherence tomography
