# Supplementary material for: Cognitive Performance and Diabetic Retinopathy: What Your Eyes Can Reveal About Your Brain
Source: Curr Diabetes Rev. 2023 Aug 2;19(9):E050822207323. doi: 10.2174/1573399819666220805154638 (PMC10617788; doi:10.2174/1573399819666220805154638)
Supplement: Supplementary file 1 [file CDR-19-E050822207323_SD1.zip › CDR-19-E050822207323_SD1/Supplementary file3_Normality tests .docx]

| Table 1: Normality Test - Baseline Characteristics – Complete Sample (n=251) | | | | | | |
| --- | --- | --- | --- | --- | --- | --- |
|  | | | **Kolmogorov-Smirnova test** | | | |
| **Variables** |  | **Statistics df** | | | **Sig.** | |
| Age (years) | .101 | | | 251 | | .000 |
| Age at Diagnosis (years) | .086 | | | 251 | | .000 |
| DM duration (years) | .121 | | | 251 | | .000 |
| Education (school years)) | .167 | | | 251 | | .000 |
| Renda Per Capta (R$ reais) | .154 | | | 241 | | .000 |
| Active time (hours/week) | .228 | | | 109 | | .000 |
| Inactive time (hours/week) | .127 | | | 237 | | .000 |
| Arterial hypertension duration (years) | .127 | | | 204 | | .000 |
| Systolic Blood Pressure (mmHg | .134 | | | 249 | | .000 |
| Diastolic Blood Pressure (mmHg) | .174 | | | 249 | | .000 |
| O2 saturation % | .179 | | | 223 | | .000 |
| BMI (Kg/m2) | .056 | | | 250 | | .059 |
| Abdominal circumference (cm) | .041 | | | 249 | | .200 |
| Neck circumference (cm) | .071 | | | 250 | | .004 |
| MMSE (score) | .158 | | | 251 | | .000 |
| Verbal fluency (score) | .082 | | | 250 | | .000 |
| TMTA (seconds) | .112 | | | 241 | | .000 |
| TMTB (seconds) | .121 | | | 207 | | .000 |
| Immediate Memory (score) | .069 | | | 229 | | .011 |
| Recall Memory (score) | .116 | | | 250 | | .000 |
| Recognition Memory (score) | .204 | | | 250 | | .000 |
| GCS(z) ^#^ | .061 | | | 251 | | .027 |
| PHQ-9 score > 9 (%) | .128 | | | 251 | | .000 |
| Urea (mg/dl) | .174 | | | 203 | | .000 |
| Creatinine (mg/dl) | .203 | | | .243 | | .000 |
| eGFR (ml/min/1.73m^2^) | .119 | | | 243 | | .000 |
| Blood glucose (mg/dl) | .138 | | | 237 | | .000 |
| HbA1c (%) | .089 | | | 239 | | .000 |
| ACR (mg/g creatinine) | .376 | | | 205 | | .000 |
| TSH (mU/L) | .053 | | | 179 | | .200 |
| Free T4 (ng/dl) | .102 | | | 204 | | .000 |
| Triglycerides (mg/dl) | .177 | | | 223 | | .000 |
| HDL cholesterol (mg/dl) | .080 | | | 222 | | .002 |
| Total cholesterol (mg/dl) | .110 | | | 222 | | .000 |
| LDL cholesterol (mg/dl) | .071 | | | 218 | | .029 |
| Vitamin B12 (pg/ml) | .145 | | | 178 | | .000 |

Note: DM: *Diabetes Mellitus*, BMI: Body mass index, MMSE: Mini Mental State Examination, TMTA: Trail Making Test A, TTB: Trail Making Test B, SCG(z): Global cognitive score(z), PHQ-9: *Patient Health Questionnaire-9,* eGFR: Estimated glomerular filtration rate, HBA1c : Glycated hemoglobin, ACR: albumin/creatinine ratio , TSH: Thyroid Stimulating Hormone, T4: Thyroxine, RAC: albumin/creatinine ratio

**Table 4: Baseline Samples Characteristics: Comparison between complete sample and sample that participated follow up**

| Characteristics | n | Complete  sample  (n = 251)  mean ±SD  %  median (IQR) | n | Follow-up Sample  (n =134)  mean ±SD  %  median (IQR) | P |
| --- | --- | --- | --- | --- | --- |
| Age (years) | 251 | 61.1 ± 9.8 | 134 | 61.5 ± 9.9 | 0.76 |
| Education (school years) | 251 | 7.6 ± 4.2 | 134 | 7.5 ± 4.3 | 0.38 |
| DM duration (years) | 251 | 12.6 ± 8.9 | 134 | 12.5 ± 8.3 | 0.87 |
| Female (%) | 251 | 56.6 | 134 | 52.2 | 0.41 |
| White Race (%) | 248 | 79.4 | 134 | 79.1 | 0.78 |
| Married / Steady Union (%) | 251 | 64.9 | 134 | 61.9 | 0.90 |
| Physically Active (%) | 251 | 27.9 | 134 | 30.6 | 0.58 |
| Smoker/Former smoker (%) | 244 | 47.1 | 134 | 42.5 | 0.39 |
| Alcoholic/Former alcoholic (%) | 244 | 29.5 | 134 | 33.6 | 0.41 |
| Diastolic Blood Pressure (mmHg) | 249 | 80.4 ±10.8 | 134 | 79.6 ± 11.3 | 0.50 |
| Systolic Blood Pressure (mmHg) | 249 | 131.2 ± 17.8 | 134 | 129.4 ± 17.8 | 0.35 |
| BMI (Kg/m2) | 249 | 30.8 ± 5.3 | 134 | 30.6 ± 4.9 | 0.48 |
| Abdominal circumference (cm) | 251 | 103.9 ± 13.1 | 134 | 103.7 ± 12.2 | 0.87 |
| Neck circumference (cm) | 250 | 39.3 ± 4.4 | 134 | 39.4 ± 4.2 | 0.82 |
| Arterial hypertension (%) | 250 | 82.4 | 134 | 79.1 | 0.43 |
| Dyslipidemia (%) | 246 | 89.0 | 134 | 92.5 | 0.28 |
| Hypothyroidism (%) | 249 | 26.1 | 134 | 28.4 | 0.63 |
| Hyperthyroidism (%) | 249 | 2.0 | 133 | 3.7 | 0.31 |
| Cardiovascular disease (%) | 250 | 35.2 | 134 | 28.4 | 0.17 |
| Diabetic Retinopathy (%) | 200 | 46.5 | 126 | 44.4 | 0.72 |
| Macular edema (%) | 196 | 13.8 | 126 | 11.1 | 0.48 |
| Diabetic Neuropathy (%) | 248 | 16.1 | 134 | 19.4 | 0.42 |
| DM kidney disease (%) | 224 | 54.0 | 130 | 47.7 | 0.25 |
| Severe hypoglycemia (%) | 240 | 21.3 | 134 | 21.6 | 0.93 |
| Depression/Anxiety | 250 | 22.8 | 134 | 17.9 | 0.26 |
| PHQ-9 score > 9 (%) | 251 | 37.1 | 134 | 39.6 | 0.63 |
| Insulin Use (%) | 250 | 58.8 | 133 | 61.9 | 0.55 |
| Statins Use (%) | 248 | 76.2 | 134 | 79.7 | 0.44 |
| Urea (mg/dl) * | 203 | 39.0(20.0) | 119 | 39.0(17.0) | 0.41 |
| Creatinine (mg/dl)* | 243 | 0.9(0.5) | 133 | 0.9(0.4) | 0.88 |
| eGFR (ml/min/1.73m^2^) ^*^ | 243 | 84.8(36.3) | 133 | 83.3(31.8) | 0.99 |
| Blood glucose (mg/dl)* | 237 | 141.0(69.5) | 132 | 147.0(71.2) | 0.73 |
| HbA1c (%)* | 239 | 8.0 (2.4) | 133 | 7.8(2.3) | 0.49 |
| ACR (mg/g creatinine) * | 205 | 21.4(80.6) | 120 | 17.7(49.6) | 0.33 |
| TSH (mU/L)* | 204 | 2.2(1.7) | 128 | 2.1(1.7) | 0.83 |
| Free T4 (ng/dl) | 179 | 1.1 ± 0.3 | 121 | 1.1± 0.3 | 0.81 |

| (continue) | | | | | |
| --- | --- | --- | --- | --- | --- |
| Triglycerides (mg/dl)* | 223 | 147.0(112.0) | 131 | 153.0(119.0) | 0.79 |
| HDL cholesterol (mg/dl)* | 222 | 42.0(14.0) | 131 | 42.0(13.0) | 0.52 |
| Total cholesterol (mg/dl)* | 222 | 160.5(54.0) | 131 | 160.0(57.0) | 0.65 |
| LDL cholesterol (mg/dl)* | 218 | 87.0(40.1) | 128 | 84.0(39.5) | 0.39 |
| Vitamin B12 (pg/ml)* | 178 | 315.5(217.0) | 119 | 340.0(286.0) | 0.39 |
| MMSE (score) | 251 | 27.2 ± 1.9 | 134 | 27.2 ± 2.0 | 0.95 |
| Verbal fluency (score) | 250 | 16.5 ± 4.9 | 133 | 16.7± 5.0 | 0.72 |
| TMTA (seconds) | 241 | 56.3 ± 27.9 | 133 | 55.3 ±26.4 | 0.72 |
| TMTB (seconds) | 207 | 162.8 ± 107.7 | 116 | 162.6 ±109.6 | 0.98 |
| Immediate Memory (score) | 229 | 16.3 ± 4.7 | 133 | 16.5 ± 4.1 | 0.76 |
| Recall Memory (score) | 250 | 5.1 ± 1.9 | 133 | 5.1 ± 1.9 | 0.74 |
| Recognition Memory (score) | 250 | 8.1 ± 2.0 | 133 | 8.4 ± 1.7 | 0.27 |
| GCS(z) (score) | 251 | -0.015 ±0.671 | 134 | 0.092± 0.631 | 0.12 |
| GCS(z) < 0 (%) | 251 | 46.6 | 134 | 41.8 | 0.36 |

*Mann Whitney Test Student -t Test for independent samples Qui-Square Test

SD: Standard deviation, IQR: Interquartil range

DM: *Diabetes Mellitus,* BMI: Body Mass Index, PHQ-9: Patient Health Questionnaire-9, eGFR: Estimated glomerular filtration rate, ACR: Albumin-to-creatinine ratio, TSH: Thyroid stimulating hormone, T4 : Thyroxine, HDL cholesterol: High density lipoprotein cholesterol, LDL cholesterol: Low Density Lipoprotein cholesterol, MMSE: Mini Mental State Exam, TMT A and B: Trial making test A and B, GCS(z): Global cognitive score (z)

**Table 5: Baseline Samples Characteristics: Comparison between sample that participated follow up and sample that did not participated follow up**

| Characteristics | n | Sample that participated follow up  (n = 117)  mean ±SD  %  median (IQR) | n | Sample that did not participated follow up  (n =134)  mean ±SD  %  median (IQR) | P |
| --- | --- | --- | --- | --- | --- |
| Age (years) | 117 | 60.8 ± 9.7 | 134 | 61.5 ± 9.9 | 0.61 |
| Education (school years) | 117 | 7.7 ± 4.2 | 134 | 7.5 ± 4.3 | 0.65 |
| DM duration (years) | 117 | 12.8 ± 9.6 | 134 | 12.5 ± 8.3 | 0.77 |
| Female (%) | 117 | 61.5 | 134 | 52.2 | 0.16 |
| White Race (%) | 114 | 78.9 | 134 | 79.1 | 0.27 |
| Married / Steady Union (%) | 117 | 68.4 | 134 | 61.9 | 0.57 |
| Physically Active (%) | 117 | 24.8 | 134 | 30.6 | 0.33 |
| Smoker/Former smoker (%) | 110 | 51.8 | 134 | 42.5 | 0.16 |
| Alcoholic/Former alcoholic (%) | 110 | 23.6 | 134 | 33.6 | 0.09 |
| Diastolic Blood Pressure (mmHg) | 115 | 81.5 ± 10.1 | 134 | 79.6 ± 11.3 | 0.17 |

| (continue) | | | | | |
| --- | --- | --- | --- | --- | --- |
| Systolic Blood Pressure (mmHg) | 115 | 133.3 ± 17.5 | 134 | 129.4 ± 17.8 | 0.85 |
| BMI (Kg/m2) | 116 | 31.1 ± 5.8 | 134 | 30.6 ± 4.9 | 0.48 |
| Abdominal circumference (cm) | 115 | 104.2 ± 14.1 | 134 | 103.7 ± 12.2 | 0.75 |
| Neck circumference (cm) | 116 | 39.2 ± 4.7 | 134 | 39.4 ± 4.2 | 0.77 |
| Arterial hypertension (%) | 116 | 86.2 | 134 | 79.1 | 0.18 |
| Dyslipidemia (%) | 114 | 85.8 | 134 | 92.5 | 0.10 |
| Hypothyroidism (%) | 115 | 22.6 | 134 | 28.4 | 0.31 |
| Hyperthyroidism (%) | 115 | 0.9 | 133 | 3.7 | 0.22 |
| Cardiovascular disease (%) | 116 | 37.1 | 134 | 28.4 | 0.17 |
| Diabetic Retinopathy (%) | 74 | 51.4 | 126 | 44.4 | 0.38 |
| Macular edema (%) | 71 | 18.3 | 126 | 11.1 | 0.20 |
| Diabetic Neuropathy (%) | 114 | 11.4 | 134 | 19.4 | 0.11 |
| DM kidney disease (%) | 94 | 62.8 | 130 | 47.7 | 0.03 |
| Severe hypoglycemia (%) | 106 | 19.8 | 134 | 21.6 | 0.75 |
| Depression/Anxiety | 116 | 27.6 | 134 | 17.9 | 0.70 |
| PHQ-9 score > 9 (%) | 117 | 33.3 | 134 | 39.6 | 0.36 |
| Insulin Use (%) | 116 | 55.2 | 133 | 61.9 | 0.30 |
| Statins Use (%) | 115 | 73.0 | 134 | 79.7 | 0.23 |
| Urea (mg/dl) * | 84 | 41.5(30.5) | 119 | 39.0(17.0) | 0.12 |
| Creatinine (mg/dl)* | 110 | 0.9(0.5) | 133 | 0.9(0.4) | 0.75 |
| eGFR (ml/min/1.73m^2^) ^*^ | 110 | 84.7(45.5) | 133 | 83.3(31.8) | 0.88 |
| Blood glucose (mg/dl)* | 105 | 134.0(70.0) | 132 | 147.0(71.2) | 0.60 |
| HbA1c (%)* | 106 | 8.2 (2.9) | 133 | 7.8(2.3) | 0.33 |
| ACR (mg/g creatinine) * | 85 | 40.4(322.0) | 120 | 17.7(49.6) | 0.49 |
| TSH (mU/L)* | 76 | 2.3(1.8) | 128 | 2.1(1.7) | 0.59 |
| Free T4 (ng/dl) | 58 | 1.1 ± 0.2 | 121 | 1.1± 0.3 | 0.46 |
| Triglycerides (mg/dl)* | 92 | 142.0(106.0) | 131 | 153.0(119.0) | 0.79 |
| HDL cholesterol (mg/dl)* | 91 | 44.0(16.0) | 131 | 42.0(13.0) | 0.65 |
| Total cholesterol (mg/dl)* | 91 | 162.0(56.0) | 131 | 160.0(57.0) | 0.52 |
| LDL cholesterol (mg/dl)* | 89 | 92.0(40.5) | 128 | 84.0(39.5) | 0.07 |
| Vitamin B12 (pg/ml)* | 59 | 297.0(118.0) | 119 | 340.0(286.0) | 0.39 |
| MMSE (score) | 117 | 27.2 ± 2.0 | 134 | 27.2 ± 2.0 | 0.89 |
| Verbal fluency (score) | 117 | 16.4 ± 4.7 | 133 | 16.7± 5.0 | 0.61 |
| TMTA (seconds) | 108 | 57.7± 29.9 | 133 | 55.3 ±26.4 | 0.50 |
| TMTB (seconds) | 91 | 164.7 ± 108.3 | 116 | 162.6 ±109.6 | 0.89 |
| Immediate Memory (score) | 117 | 16.1 ± 4.9 | 133 | 16.5 ± 4.1 | 0.56 |
| Recall Memory (score) | 117 | 5.0 ±2.0 | 133 | 5.1 ± 1.9 | 0.56 |
| Recognition Memory (score) | 117 | 7.9 ± 2.3 | 133 | 8.4 ± 1.7 | 0.05 |
| GCS(z) (score) | 117 | -0.062 ± 0.689 | 134 | 0.092± 0.631 | 0.06 |
| GCS(z) < 0 (%) | 117 | 47.0 | 134 | 41.8 | 0.45 |

*Mann Whitney Test Student -t Test for independent samples Qui-Square Test

SD: Standard deviation. IQR: Interquartil range

DM: *Diabetes Mellitus,* BMI: Body Mass Index, PHQ-9: Patient Health Questionnaire-9, eGFR: Estimated glomerular filtration rate, ACR: Albumin-to-creatinine ratio, TSH: Thyroid stimulating hormone, T4: Thyroxine, HDL cholesterol: High density lipoprotein cholesterol, LDL cholesterol: Low Density Lipoprotein cholesterol, MMSE: Mini Mental State Exam, TMT A and B: Trial making test A and B, GCS(z): Global cognitive score (z)
