# Supplementary material for: Cognitive Performance and Diabetic Retinopathy: What Your Eyes Can Reveal About Your Brain
Source: Curr Diabetes Rev. 2023 Aug 2;19(9):E050822207323. doi: 10.2174/1573399819666220805154638 (PMC10617788; doi:10.2174/1573399819666220805154638)
Supplement: Supplementary file 1 [file CDR-19-E050822207323_SD1.zip › CDR-19-E050822207323_SD1/Supplementary file4_Detailed sample characteristics.docx]

| Table 1: Detailed Sample Characteristics | | | | |
| --- | --- | --- | --- | --- |
| Variáveis | N | % /  Média ± DP | Mediana | IIC |
| **Age** $\boldsymbol{\geq}$ **65 (years) (%)** | 251 | 42.2 |  |  |
| **Age (years)** | 251 | 61.1 ± 9.8 | 63.0 | 13.0 |
| **Age at Diagnosis (years)** | 251 | 48.5 ± 11.3 | 50.0 | 17.0 |
| **Gender (%)**  Female  Male | 251 | 56.6  44.4 |  |  |
| **Race (%)**  White  Black  Mulato  Asiatic  Indian | 248 | 79,4  4,4  14,9  1,2  0,0 |  |  |
| **Marital state (%)**  Married / Stable union  Divorced  Single  Widowed | 251 | 64.9  15,5  9,2  10,4 |  |  |
| **Renda Per Capta ($Reais)** | 241 | 1095.27 ± 263.65 | 1000.00 | 783.33 |
| **Schooling (school years)** | 251 | 7.6 ± 4.2 | 7.0 | 7.0 |
| **Schooling** $\boldsymbol{\leq}$ **6 (school years) (%)** | 251 | 47.0 |  |  |
| **Smoker/Former smoker (%)** | 244 | 47.1 |  |  |
| **Alcoholic/Former alcoholic (%)** | 244 | 29.5 |  |  |
| **Physically Active (%)** | 251 | 27.9 |  |  |
| **Active time (hours/week)** | 237 | 27.7 ± 20.8 | 22.0 | 23.0 |
| **Inactive time (hours/week)** | 109 | 4.5 ± 4.6 | 3.3 | 3.0 |
| **DM duration (years)** | 251 | 12.6 ± 8.9 | 10.0 | 14.0 |
| **DM duration ≥ 10 (years) (%)** | 251 | 59.4 |  |  |
| **Arterial hyperetension (%)** | 250 | 82.4 |  |  |
| **Arterial hypertension duration (years)** | 204 | 15.1 ± 10.8 | 13.0 | 13.0 |
| **Dyslipidemia (%)** | 246 | 89.0 |  |  |
| **Hypothyroidism (%)** | 249 | 26.1 |  |  |
| **Hyperthyroidism (%)** | 249 | 2.0 |  |  |
| **Cardiovascular disease (%)**  Myocardial revascularization  Angina pectoris  Heart failure  Acute myocardial infarction  Peripheral arterial disease | 250 | 35.2  24.4  5.2  16.8  14.5  8.4 |  |  |
| Diabetic Retinopathy (%)  RNP  RP | 200 | 46.5  35.0  11.5 |  |  |
| **Macular edema (%)** | 196 | 13.8 |  |  |
| **Diabetic Neuropathy (%)** | 248 | 16.1 |  |  |
| **DM kidney disease (%)** | 224 | 54.0 |  |  |
| **Severe hypoglycemia (%)** | 240 | 21.3 |  |  |
| **Depression/Anxiety (%)** | 250 | 22.8 |  |  |
| **Insulin use (%)** | 250 | 58.8 |  |  |
| **Statin use (%)** | 248 | 76.2 |  |  |
| **PHQ-9 (score) (%)** | 251 | 8.7 ±7.0 | 7.0 | 10.0 |
| **Escore PHQ-9 > 9** | 251 | 37.1 |  |  |
| **Diastolic Blood Pressure (mmHg)** | 249 | 80.4 ± 10.8 | 80.0 | 20.0 |
| **Systolic Blood Pressure (mmHg)** | 249 | 131.2 ± 17.8 | 130.0 | 20.0 |
| **BMI (Kg/m2)** | 249 | 30.8 ± 5.3 | 30.2 | 8.2 |
| **Abdominal circumference (cm)** | 251 | 103.9 ± 13.1 | 104.0 | 17.5 |
| **Neck circumference (cm)** | 250 | 39.3 ± 4.4 | 39.0 | 7.0 |
| **O2 saturation %** | 223 | 95.3 ± 2.6 | 96.0 | 3.0 |
| **MMSE (score)** | 251 | 27,2± 2,0 | 28,0 | 3,0 |
| **Verbal fluency (score)** | 250 | 16,5 ± 4,9 | 16,0 | 7,0 |
| **TMTA (seconds)** | 241 | 56,3± 27,9 | 52,0 | 36,0 |
| **TMTB (seconds)** | 207 | 162,8 ± 107,7 | 138,0 | 130,0 |
| **Immediate Memory (score)** | 229 | 16,3 ± 4,5 | 16,0 | 6,0 |
| **Recall Memory (score)** | 250 | 5,1 ±1,9 | 5,0 | 2,0 |
| **Recognition Memory (score)** | 250 | 8,1 ± 2,0 | 9,0 | 3,0 |
| **GCS(z) (score)** | 251 | -0,015 ± 0,671 | 0,052 | 1,013 |
| **GCS(z) < 0** | 251 | 46.6 |  |  |
| **Urea (mg/dl)** | 203 | 45,8 ± 23,0 | 39,0 | 20,0 |
| **Creatinine (mg/dl)** | 243 | 1,0 ± 0,6 | 0,9 | 0,48 |
| **eGFR (ml/min/1.73m^2^)** | 243 | 77,3 ± 25,6 | 84,8 | 36,3 |
| **Fasting blood glucose (mg/dl)** | 237 | 156,3 ± 70,9 | 141,0 | 69,5 |
| **HbA1c (%)** | 239 | 8,2 ± 1,9 | 8.0 | 2,4 |
| **ACR (mg/g creatinine)** | 205 | 246,8 ± 646,8 | 21,4 | 80,6 |
| **TSH (mU/L)** | 204 | 2,4 ± 1,4 | 2,2 | 1,7 |
| **Free T4 (ng/dl)** | 179 | 1,1± 0,3 | 1,1 | 0,34 |
| **Triglycerides (mg/dl)** | 223 | 183,3 ± 141,9 | 147,0 | 112,0 |
| **HDL cholesterol (mg/dl)** | 222 | 44,7 ±13,5 | 42,0 | 20,0 |
| **Total cholesterol (mg/dl)** | 222 | 166,9 ± 44,8 | 160,5 | 0,48 |
| **LDL cholesterol (mg/dl)** | 218 | 90,4 ± 35,4 | 87,0 | 36,3 |
| **Vitamin B12 (pg/ml)** | 178 | 375,3 ± 200,3 | 315,5 | 69,5 |

SD: Standard deviation, IQR: Interquartil range

DM: *Diabetes Mellitus,* BMI: Body Mass Index, PHQ-9: Patient Health Questionnaire-9, MMSE: Mini Mental State Exam, TMT A and B: Trial making test A and B, GCS(z): Global cognitive score (z),, eGFR: Estimated glomerular filtration rate, ACR: Albumin-to-creatinine ratio, TSH: Thyroid stimulating hormone, T4: Thyroxine, HDL cholesterol: High density lipoprotein cholesterol, LDL cholesterol: Low Density Lipoprotein cholesterol.
