# Supplementary material for: Cognitive Performance and Diabetic Retinopathy: What Your Eyes Can Reveal About Your Brain
Source: Curr Diabetes Rev. 2023 Aug 2;19(9):E050822207323. doi: 10.2174/1573399819666220805154638 (PMC10617788; doi:10.2174/1573399819666220805154638)
Supplement: Supplementary file 1 [file CDR-19-E050822207323_SD1.zip › CDR-19-E050822207323_SD1/Supplementary file6_Multivariate Logistic regression.docx]

**Table 1: Baseline Multivariate Binary Logistic Regression Model - GCS < 0 and explanatory variables***

| Variable | Classification | N | GCS 1 < 0  n (%) | P | Odds Ratio (CI 95%) |
| --- | --- | --- | --- | --- | --- |
| **Age**  **(years)** | $\geq$ 65 | 106 | 67 (57.3) | 0.000 | 5.461 (2.417– 12.344) |
|  | $<$ 65 | 145 | 50 (42.7) |  |  |
| **School education**  **(years)** | $\leq$ 6 | 166 | 87 (74.4) | 0.000 | 12.192 (5.618 – 26.459) |
|  | $>$ 6 | 133 | 30 (25.6) |  |  |
| **Arterial hypertension** | Yes | 206 | 106 (90.6) | 0.086 | 2.545 (0.877 – 7.389) |
|  | No | 44 | 11 (9.4) |  |  |
| **PHQ-9 s > 9** | Yes | 93 | 50 (42.7) | 0.003 | 3.533 (1.546 – 8.075) |
|  | No | 158 | 67 (57.3) |  |  |
| **Diabetic retinopathy** | Yes | 93 | 53 (58.2) | 0.017 | 2.505 (1.176 – 5.340) |
|  | No | 107 | 38 (41.8) |  |  |

Note: Method Backward conditional

*Variables included: age ≥ 65 years, ≤ 6 school years, Diabetes duration ≥ 10 years, physical activity, PHQ-9 score > 9, arterial hypertension, cardiovascular disease, diabetic retinopathy, macular edema.

GCS = Global cognitive score (z)
